# Midwives of Old

**Published:** 1890-06-21

**Authors:** 


### Midwives of Old.

The project now under consideration for the "education, licensing, and registration" of midwives is an excellent but not a new one; if carried out, it will be the revival of a system which prevailed generally in England throughout the middle ages, and which lingered on in Scotland and Ireland till a comparatively recent date, as witness the following certificate, granted by Dr. Young in 1758: "These are to certify that Mrs. Hope, midwife, attended three courses of my lectures upon the theory and practice of midwifery, by which means she had an opportunity of seeing and operating in all the different sorts of births. As witness my hand and seal at Edin. this 23d day of June, 1758. Thomas Young, Professor of Midwifery in the University of Edin."

Fashion, as to the sex of the person selected to attend childbirths, has undergone a good many changes. Agnodice, the Athenian maiden who had learnt the art of midwifery from her father, and who desired to practise it, had to disguise her sex before she could get patients; but once "in practice," her example was followed by other women, and the male practitioner of midwifery was gradually blotted out. The Scriptural allusions to midwifery show very clearly that amongst the Hebrews and Egyptians, women were the sole practitioners of the art. So in England throughout the centuries for which records exist, the allusions to the births of distinguished persons invariably show that their mothers were attended in childbirth entirely by women—a fact in which may, perhaps, lie the origin of the troublesome importance which the modern "monthly nurse"—the survival of the ancient midwife—always attaches to her office. No doubt the midwife of old was a person of some social importance, coming as she did, when necessity occasioned her visit, into the house with her servants and attendants, who themselves seem to have been termed "wives." An interesting book of household payments, compiled by a Norfolk squire some four centuries back, contains the record of a fee given "Toe my mistresses mydwyffe and her wyffes, ye whole week." Was a week the usual length of the midwife's visit? Occasional entries in old documents (rentals and the like) lead one to conjecture that each town possessed its one or more midwives, according to its population; for instance, in 1547 we find a house in Birmingham described as being in the occupation of "the mydwyffe" of that town.

In early times the midwife's licence came from the Bishop of the diocese in which she intended to practice—at the beginning of the last century it cost a little under £1 to obtain such licence. A solemn oath had to be taken by the would-be practitioner, which wrung from her not so much a declaration of her medical skill as a promise not to ally herself with the demoniacal powers in carrying on her work. One form of oath ran as follows: "You shall not anywise use or exercise any manner of witchcraft, charm, or sorcery, invocation, or other prayers than may stand with God's laws and the king's." The rite of baptism, in cases where a new-born infant was thought unlikely to survive its birth long enough to obtain the services of a priest, was by ecclesiastical authority specially entrusted to the midwife. Many parish registers in the country contain entries of such baptisms, and in an old charge given by the Archbishop of York to his clergy we read: "Item. All curates must, openly in the churche, teeche and instructe the mydwiefs of the very wordes and fourme of baptisme to th'entente they maye knowe them perfectly, and none oder." In a dispute that arose in 1523 as to some property belonging to the Everey family, Elizabeth Gaynsford, the midwife, deposes: "I, the aforesaid Elizabeth, seeing the child of Thomas Everey, late born, in jeopardy of life, by authority of my office, then beyng midwyfe, did christen the same child under this manner: In the name of the Fader, the Son, and the Holy Ghost, I christen thee Denys, at the same time throwing unconsecrated water."

The College of Physicians was founded in 1518, and very soon afterwards there are indications that midwifery was coming to be regarded as a science worthy the attention of male practitioners. Eucharius Rhodion devoted his energies to the publication of a work on the subject, which Dr. Raynolds translated into English, and published in 1540 under the title of "The Byrthe of Mankind, otherwise named the Woman's Booke." Many eminent medical men then announced themselves practitioners of the art, and after the famous Dr. Harvey had followed it, in the early years of the seventeenth century, the prospects of the female midwives looked gloomy enough, for though in the country, and with certain classes of the population, the antipathy to male practitioners was as intense as in very ancient times had been that towards women, in the large towns and amongst the wealthy and educated, the male "midwives" became exceedingly popular.

To obtain a recognized social standing, the female midwives endeavoured to get established as a "company" or "mystery": the first we hear of this is in 1616. The College of Physicians set itself resolutely against the proposal, and it was dropped for the time. However, in or about the year 1634, Dr. Peter Chamberlain—himself extensively engaged in the practice of midwifery—laboured, in direct opposition to the wishes of the college, to organize the female midwives into a company, with himself as their head, and as their director and "examiner." But the opposition of the college prevailed, and the useful scheme fell through. However, the result was not to drive the female practitioners wholly out of the field, though the refusal of the college prevented much of their utility by refusing to grant them certificates of medical capacity, and the midwives still continued under their former ecclesiastical certificates. A curious letter, written in 1737 by a surgeon at Manchester who was not getting on so well as he desired, expresses regret that he had not contented himself with a practice offered him at Leicester, "for," says he, "there is not a man-widwife within ten miles of that town." In Ireland the male-midwife seems to have been by no means a popular institution so late even as the middle of the last century, when Dr. Van Lewen became an eminent practitioner of the art; he was then induced to take it up, says his daughter, because there was then [1740 *circa*] "but one man-midwife in the kingdom." Whether the female midwives in Ireland held any certificate from Trinity College we do not know, but in Scotland we have seen that in 1758 such certificates were granted by the Edinburgh professor of midwifery on the same lines as it is now proposed to bestow them in England. In giving the Edinburgh midwives their diplomas, Dr. Young seems to have said these words of caution: "You are not to expect that in your sphere of education you can ever be qualified to practise physic. You may do service in slight disorders, where gentlemen of liberal education are not, or cannot, be consulted," but, he continues, "be not so foolhardy" as in difficult cases to rely "on your own judgment." An old Scotch midwife, in giving evidence five and thirty years ago, speaks of having had some 1,565 cases in 60 years' practice; so the popularity of female midwives in Scotland must have lingered on well into this century.

The midwives of old seem occasionally to have been called upon to perform duties somewhat outside what would appear to be the proper sphere of their operations. In 1634 the Privy Council wrote to certain of his Majesty's surgeons directing them "to make choice" of such midwives as they may think fit "to inspectt and search the boddies of those women that were latlie brought up by the Sheriff of the County of Lancaster, indicted for witchcrafte," and to report as to any "unnatural marks" which they discover. The prisoners were then at the "Shippe Taverne at Greenwiche." The report is extant. It gives a curious picture of the method of proceeding, and concludes by stating that any "unusual" marks found upon the suspected witches were easily accountable for by natural causes, a report which, we remember, did not secure the acquittal of some of the unfortunate women indicted.

